# Cervical Lymph Node Metastasis From Medulloblastoma in a Young Adult: Case Report and Literature Review

**DOI:** 10.7759/cureus.61339

**Published:** 2024-05-29

**Authors:** Mariam Harrak, Saiss Kamal, Hamza Zerbani, Hajar El Bakouri, Saoussan Ouaya, Nabila Sellal, Mohamed El Hfid

**Affiliations:** 1 Radiation Therapy Department, University Hospital Center Mohamed VI of Tangier, Tangier, MAR; 2 Pathology and Laboratory Medicine, Avenzoar Pathological Anatomy Center, Tangier, MAR

**Keywords:** brain tumor, radiotherapy, cervical lymph node metastasis, extraneural metastasis, medulloblastoma

## Abstract

Medulloblastoma, an embryonal tumor located in the posterior fossa of the brain, originates from the neuro-epidermal layer of the cerebellum. It is the most prevalent malignant tumor in children, while it is rare in adults and predominantly affects males. Multimodal therapeutic interventions, such as surgery, radiotherapy, and chemotherapy, have substantially enhanced the prognosis of this condition. Extraneural metastases are infrequent. We present a case of medulloblastoma relapse with nodal metastasis in a 28-year-old adult.

## Introduction

Cushing and Bailey first described medulloblastoma in 1925 as an embryonal tumor of the posterior fossa of the brain. It arises from the neuroepithelial layer of the cerebellum [1]. It is the most common malignant central nervous system tumor in children and is extremely rare in adults. It is characterized by a male predominance [2,3,4]. Multimodal approaches combining surgery, radiotherapy, and chemotherapy have improved the prognosis of medulloblastoma. The survival rates are 79.9% at two years, 64.9% at five years, and 52.1% at 10 years [5].

We present a case of nodal metastatic relapse of medulloblastoma in a 28-year-old adult, and through this observation, we discuss the prognosis and evolution of this cancer.

## Case presentation

**Figure 1:**
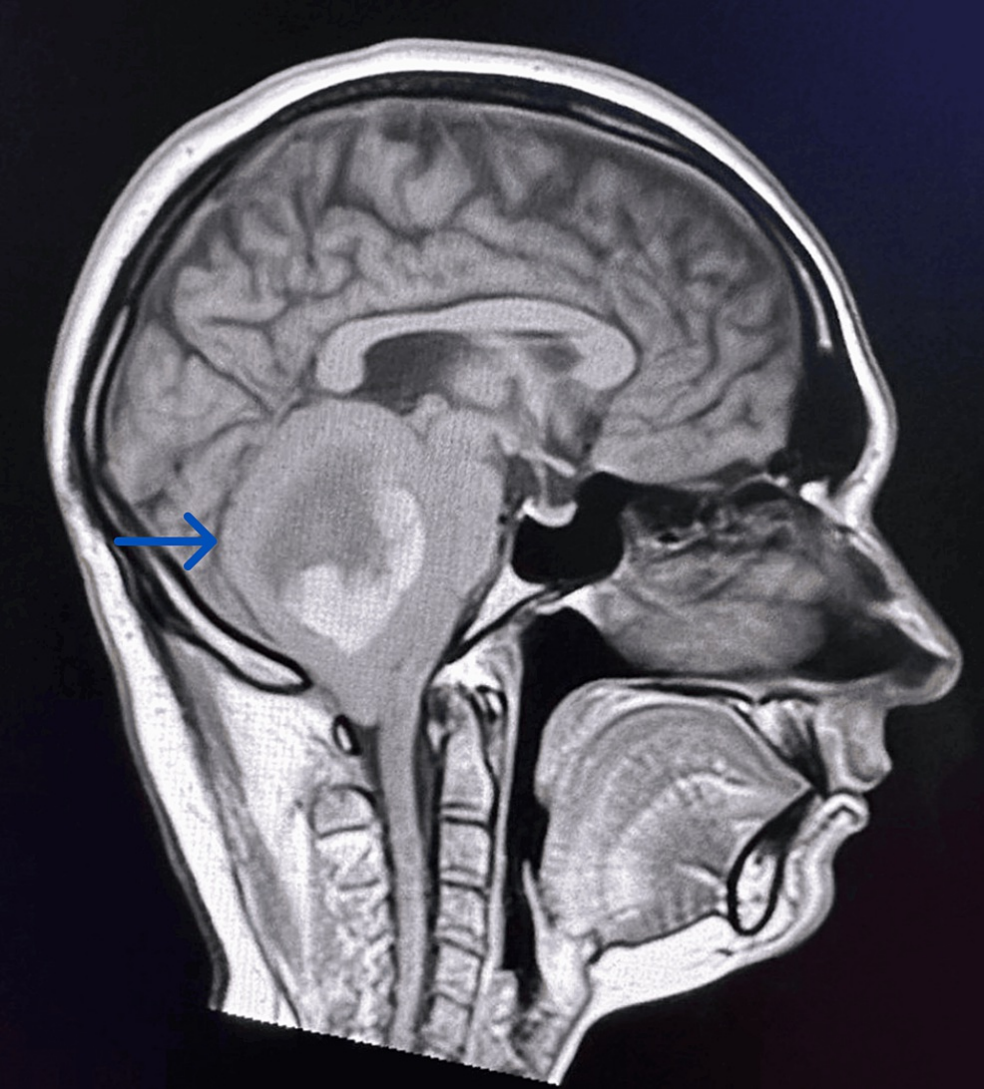
Initial diagnostic magnetic resonance imaging head scan of the patient with axial T1 contrast views demonstrating a cerebral lesion.

This concerns a 28-year-old patient who underwent treatment around five years ago for a medulloblastoma, which presented with symptoms of intracranial hypertension and cerebellar syndrome. Preoperative magnetic resonance imaging (MRI) revealed a large intra-axial lesion at the right cerebellopontine angle with a cystic lesion measuring 49 mm × 42 mm, exerting a mass effect on the fourth ventricle (Figure 1).

**Figure 2:**
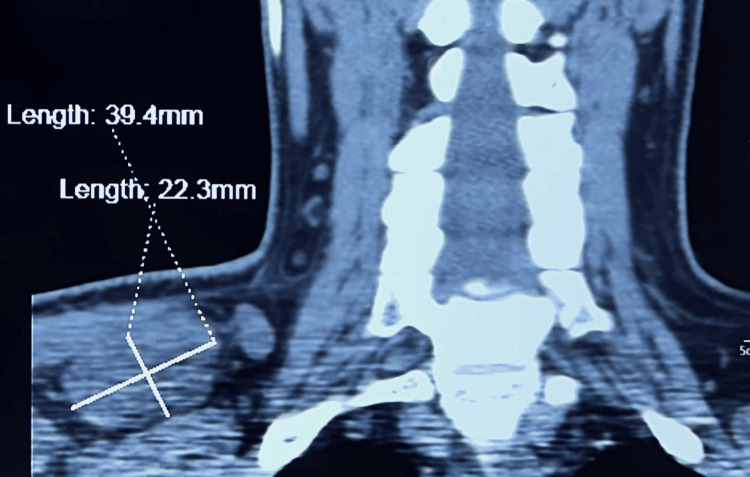
Cervical-thoracic CT scan performed 36 months after treatment revealing a right supraclavicular mass.

The patient underwent total tumor resection with ventriculoperitoneal shunting. Postoperative craniospinal irradiation was performed at a dose of 30 gray to the craniospinal axis and a boost of 24 gray to the tumor bed. Thirty-six months after treatment completion, the patient presented with right cervicobrachial neuralgia. A clinical examination revealed a hard and painful right supraclavicular mass. The cervical-thoracic computed tomography scan (CT scan) revealed a right supraclavicular mass measuring 39.4 mm × 49.7 mm. Craniospinal MRI showed no signs of local recurrence (Figure 2).

**Figure 3:**
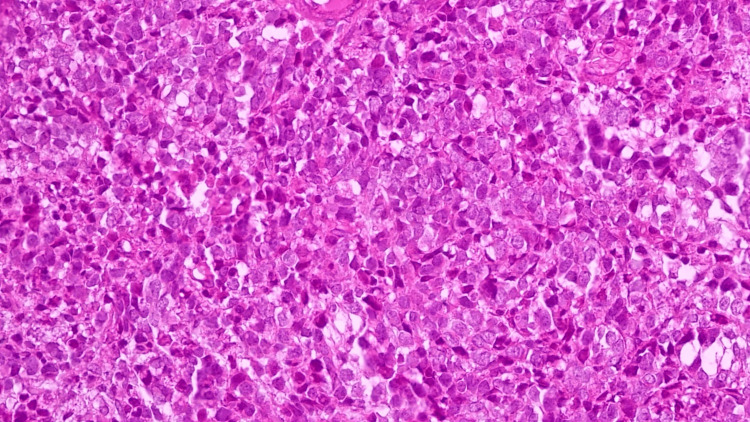
Histological examination of the biopsy of the supraclavicular mass: small cells were with limited cytoplasm, and hyperchromatic nuclei. Immunohistochemical examination: CD56+, Synaptophysin+, GFAP+, P53, and Ki67+ antibodies, Cytokeratin-, CD99-, and Neurofilament-.

Histopathological and immunohistochemical analysis of a biopsy from this mass revealed malignant tumor proliferation consistent with a supraclavicular lymph node metastasis of medulloblastoma. The tumor cells were small, with scant cytoplasm and hyperchromatic nuclei. Immunohistochemical analysis showed expression of CD56 (cluster of differentiation 56), synaptophysin, GFAP (glial fibrillary acidic protein), P53 (tumor protein 53), and Ki67 antibodies in the sampled cells. However, cytokeratin, CD99 (cluster of differentiation 99), and neurofilament antibodies were all negative (Figure 3).

The mass was not surgically resectable, and primary chemotherapy followed by right supraclavicular irradiation was decided upon in a multidisciplinary meeting. The patient received three cycles of PCV (procarbazine, lomustine, and vincristine)-type primary chemotherapy, resulting in significant clinical improvement. Evaluation scanning showed a 60% reduction in the initial mass size.

Subsequently, the patient underwent radiotherapy at the recurrence site using three-dimensional techniques. He received a dose of 54 gray with a conventional fractionation of 2 gray per session. While undergoing radiotherapy, grade I radiodermatitis was observed at the supraclavicular fossa. After 10 months of follow-up, the patient remains in complete clinical and radiological remission.

## Discussion

Medulloblastoma is a highly malignant tumor of the cerebellum and one of the most common brain tumors in children. It frequently metastasizes along the pathways of the cerebrospinal fluid. However, extraneural metastases are considered rare [6,7]. The blood-brain barrier and the presumed absence or lack of lymphatic vessels are thought to be the causes of the low incidence of metastases outside the central nervous system [8].

Eberhart et al. [9] reported the cases of 23 patients diagnosed with medulloblastoma with systemic metastases. The most common sites were bone and bone marrow (91%). The infiltration of other soft tissues and/or lymph nodes was very rare (13%). Thirty percent of patients had multiple metastatic sites. In our case, the right supraclavicular area was the only metastatic site since the extension assessment did not show any other metastatic locations.

Varan et al. published a study on the extraneural metastasis of brain tumors in children. Reported extraneural metastasis accounted for 0.98% of 1011 patients treated for brain tumors, of which six cases were medulloblastomas, and two of them presented with cervical lymph node metastasis [7].

According to a systematic review of the literature published between 1944 and 2021 on metastasis to cervical lymph nodes from central nervous system tumors (including data from 143 articles and involving 174 patients), the most common distant metastatic sites were bone (23%), lungs (11.5%), and lymph nodes (11%). Based on all the publications mentioned earlier, the predominant sites of systemic metastases were bone and bone marrow. However, metastases to soft tissues, lymph nodes, or lungs are much rarer [10] (Table 1).

**Table 1:** Table showing the most common sites of medulloblastoma metastases.

References	Number of cases	Average age	Metastatic sites
Andrés Coca-Pelaz et al. [10-16]	27	8.9 (0.6–31)	10 cases of bone metastasis
			3 cases of lung metastasis
			3 cases of lymph node metastasis
			2 cases of skin metastasis
			1 case of liver metastasis
			1 case of spine metastasis
Eberhart et al. [9]	23	8.5 (1–40)	10 cases of bone metastasis
			11 cases of bone marrow metastasis
			2 cases of lymph node metastasis, including 1 case in the cervical site
			1 case of lungs metastasis
Varan et al. [7]	6	9.38 (3.4–15)	2 cases of bone metastasis
			2 cases of lungs metastasis
			2 cases of cervical lymph nodes metastasis
			1 case of bone marrow metastasis
			1 case of liver metastasis
			1 case of nasal sinuses metastasis

Given the presumed absence of lymphatic vessels within the central nervous system, the mechanism by which central nervous system tumors spread to lymph nodes remains incompletely understood. Several authors have attempted to explain why primary brain tumors may metastasize to cervical lymph nodes. Among the hypotheses found in the literature are: Yağmurlu et al. described the connection between deep cervical lymph nodes and lymphatic vessels with the intracranial space through the jugular foramen in humans [17,18]. Surgical interventions mechanically disrupt the efficacy of the blood-brain barrier as a limit against the migration of tumor cells [10]. The reported median time for the appearance of systemic metastases ranged from 12 to 32 months. This timeframe can exceed five years in some cases [19,20]. In our patient's case, this interval was 36 months. Cases of cervical lymph node metastases from medulloblastoma are rare, making it challenging to standardize their treatment. Therapeutic regimens for extraneural metastasis of medulloblastoma included surgical resection followed by chemotherapy, chemotherapy combined with radiotherapy, chemotherapy alone, or radiotherapy alone. The choice of therapeutic strategy depends primarily on the site of the metastasis, its resectability, and the patient's condition. The literature suggests performing either total or subtotal surgical resection if possible. If surgery is not feasible, local radiotherapy may be an option [10͵20].

## Conclusions

Metastasis of medulloblastoma outside the central nervous system is rare and may occur after long periods of clinical remission. The most common sites of metastasis are bones and bone marrow, followed by lymph nodes and, to a lesser extent, the liver, lungs, and peritoneum. The mechanism of these relapses is not yet fully understood, and therapeutic management is not standardized, underscoring the need for broader studies.
